# Identification of acetic acid sensitive strains through biosensor-based screening of a *Saccharomyces cerevisiae* CRISPRi library

**DOI:** 10.1186/s12934-022-01938-7

**Published:** 2022-10-15

**Authors:** Maurizio Mormino, Ibai Lenitz, Verena Siewers, Yvonne Nygård

**Affiliations:** Department of Biology and Biological Engineering, Chalmers University of Technology, Gothenburg, Sweden

**Keywords:** Acetic acid, Biosensor, Library, Screening, Tolerance, CRISPRi, Yeast

## Abstract

**Background:**

Acetic acid tolerance is crucial for the development of robust cell factories for conversion of lignocellulosic hydrolysates that typically contain high levels of acetic acid. Screening mutants for growth in medium with acetic acid is an attractive way to identify sensitive variants and can provide novel insights into the complex mechanisms regulating the acetic acid stress response.

**Results:**

An acetic acid biosensor based on the *Saccharomyces cerevisiae* transcription factor Haa1, was used to screen a CRISPRi yeast strain library where dCas9-Mxi was set to individually repress each essential or respiratory growth essential gene. Fluorescence-activated cell sorting led to the enrichment of a population of cells with higher acetic acid retention. These cells with higher biosensor signal were demonstrated to be more sensitive to acetic acid. Biosensor-based screening of the CRISPRi library strains enabled identification of strains with increased acetic acid sensitivity: strains with gRNAs targeting *TIF34*, *MSN5*, *PAP1*, *COX10* or *TRA1*.

**Conclusions:**

This study demonstrated that biosensors are valuable tools for screening and monitoring acetic acid tolerance in yeast. Fine-tuning the expression of essential genes can lead to altered acetic acid tolerance.

**Supplementary Information:**

The online version contains supplementary material available at 10.1186/s12934-022-01938-7.

## Background

In the past decades, a vast range of products, including biofuels, bulk and fine chemicals, nutraceuticals and pharmaceuticals have been produced using microbial cell factories. Biotechnological production of industrially relevant products from biomass unsuited as food or feed is considered a viable replacement for current petroleum-based products. Still, a challenge in using these so-called lignocellulosic biomasses as a raw material is that they often contain compounds that are inhibitory for the cell factories, namely furfural, weak acids and phenols [1]. Among these compounds, acetic acid formed during hydrolysis is one of the most limiting factors when using baker´s yeast *Saccharomyces cerevisiae* as a cell factory for conversion of lignocellulosic biomass into biochemicals [2]. Tolerance towards acetic acid in yeast is achieved through a set of complex mechanisms [3] and acetic acid tolerance still represents a major bottleneck for the development of second generation biorefineries [4] where lignocellulosic biomass is used as a raw material. A great amount of work has been done on engineering acetic acid tolerance in yeast through e.g. deletion or overexpression of specific genes [5] but rational strain engineering for increased acetic acid tolerance remains challenging [6]. Moreover, the genetic background of the strain may critically influence the effect of a gene alteration, which constitutes a great challenge for rational strain engineering [5].

In the past few years the CRISPR interference (CRISPRi) technology has arisen as an efficient tool for altering gene expression [7]. This technology is based on a CRISPR-associated protein, often an endonuclease-deficient version of Cas9, (dCas9; dead Cas9) targeted to the promoter region of a gene, using a guide RNA (gRNA). At the promoter, dCas9 can interfere with the endogenous transcription machinery, and this way downregulate the expression of a gene [8]. The level of downregulation is dependent on the targeting site and can be enhanced by attaching a repressor to dCas9 [9] or to the gRNA [10]. The CRISPRi technology has successfully been used for fine-tuning gene expression and allows downregulation of essential genes by conditionally expressing *dCas9* [11] or the gRNA [12]. Using this approach, several CRISPRi strain libraries targeting a large part or even all of the genes of *S. cerevisiae* have been constructed [13–17].

Screening of large strain libraries containing systematically designed mutants is an important tool for fundamental cell biology. This may allow the identification of new nonintuitive engineering targets and expand our knowledge over the circuits regulating the cell. Similarly, screening and identifying improved mutants generated through untargeted approaches such as mutagenesis or adaptive evolution can improve our understanding of what gives a strain a fitness benefit. In recent work, screening CRISPRi strain libraries has led to the identification of novel genes involved in tolerance towards inhibitory compounds [13, 15, 18]. In the screen by Gutmann et al. [13], *HAA1*, *STB5* and *YAP1* were targeted in strains identified as sensitive to inhibitors found in lignocellulosic hydrolysates. These three genes have previously been shown to increase tolerance when overexpressed. Similarly, *GLC7*, that when repressed led to strong acetic acid sensitivity [18] was showed to improved acetic acid tolerance when overexpressed [84]. Thus, genes of sensitive CRISPRi mutants are plausible targets for improving tolerance through overexpression. The earlier CRISPRi screens were conducted using competitive growth followed by next generation sequencing [12, 15, 16], immunostaining followed by cell sorting [15], or through measuring the growth of the individual strains in the phenomics platforms Scan-o-matic [18]. Still, the screening of large strain libraries represents a bottleneck, especially when the desired outcome does not result in an easy-to-monitor phenotype [19].

Biosensors are efficient tools for screening strain libraries or monitoring strain performance [19], including production of specific compounds [20–23], intracellular pH [24, 25], various stress responses [24, 26] or concentration of compounds such as metals [27], sugars [28] or intracellular metabolites, such as ATP [29], fructose-bisphosphate [30] or malonyl-CoA [31]. The use of biosensors can considerably accelerate strain evaluation and can also allow real-time monitoring of cellular states [19].

Transcription factor (TF) based biosensors are among the most commonly used biosensors; these typically convert the signals of a specific compound into a readout such as expression of a fluorescent protein [32]. The expression of the biosensor reporter can be translated to describe the concentration of the target molecule. Commonly, yeast biosensors exploit heterologous prokaryotic TFs [19], but endogenous eukaryotic TFs have also been successfully used for monitoring the NADPH/NADP^+^ ratios [33] or sensing acetic acid [22]. Several parameters may describe the performance of a biosensor, including specificity, sensitivity, dynamic and operational range. The properties of TFs as well as promoters have been showed to impact those features [19]. In particular, the dynamic and operational range of a biosensor can be increased by varying the amount of binding sites for the TF in the promoter driving the reporter [34, 35].

We recently described the design, characterization and application of a TF-based biosensor reporting acetic acid production in *S. cerevisiae* [22]. This biosensor is based on the endogenous zinc-finger TF Haa1 which has been reported to relocate from the cytoplasm to the nucleus upon direct binding of acetate [36, 37]. In this biosensor, we fused Haa1 with the C-terminus of the bacterial DNA-binding protein BM3R1 [38] and the N-terminus of the cyan fluorescent protein mTurquoise2, resulting in the synthetic TF (sTF) BM3R1-Haa1-mTurquoise2. The biosensor readout is expression of the red fluorescent protein mCherry, under the control of a synthetic promoter that includes binding sites for the BM3R1 DNA-binding protein enclosed in the biosensor. In Mormino *et al.* [22] we demonstrated that this biosensor was able to report both acetic acid added to the medium and acetic acid produced by the cells themselves.

In this study, we improved the dynamic range of the acetic acid biosensor and used it to screen a *S. cerevisiae* CRISPRi strain library [17] for tolerance towards acetic acid. Genes encoding the acetic acid biosensor components and the pH-sensitive GFP variant sfpHluorin [39] were integrated in the genome of the library strains. This allowed monitoring acetic acid retention and intracellular pH of strains identified in the screen. The screen allowed us to identify a set of strains with increased sensitivity towards acetic acid stress and suggests novel gene targets for increasing acetic acid tolerance.

## Results

### Novel biosensor design and biosensor characterization in the CRISPRi library strains

**Fig. 1:**
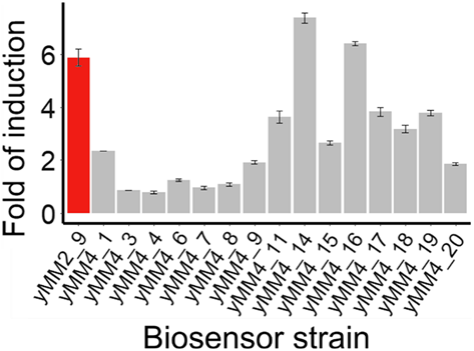
Fold of induction of the different biosensor variants. The ratio between the maximal reporter signal observed at 50 mM acetic acid compared to the fluorescence level of the same strain at 0 mM acetic acid is shown. The original biosensor containing strain yMM2_9 is highlighted in red. Data were obtained from three biological replicates; whiskers show the standard deviation

To improve the dynamic range of the acetic acid biosensor described in Mormino *et al.* [22], 15 promoters of varying strength were tested for expression of the sTF open reading frame (ORF) *BM3R1-HAA1-mTurquoise2*. All promoters except *TEFmut7*, a modified, constitutive version of the *TEF1* promoter [85], are part of the MoClo collection [79]. The reporter expression and growth of strains were monitored over time at 0 and 50 mM acetic acid (Fig. 1; Additional file 1: Fig. S1). The greatest dynamic range was obtained with the strain expressing *BM3R1-HAA1-mTurquoise2* under the *RET2* promoter (yMM4_14), improving the dynamic range of the biosensor by approx. 20%.

**Fig. 2:**
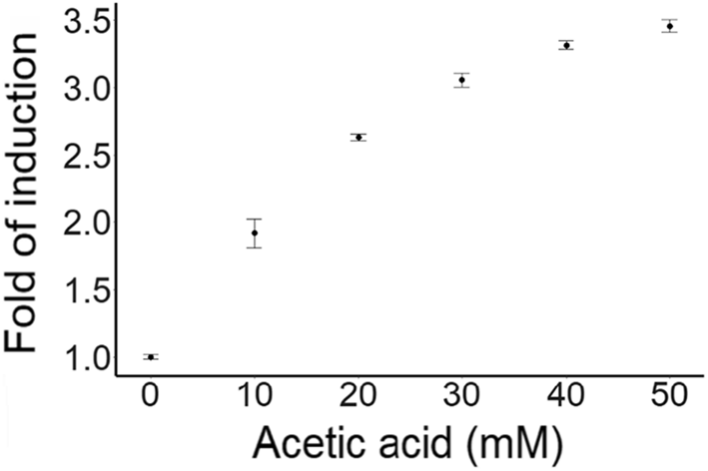
The fold of induction of the biosensor at varying concentrations of acetic acid and pH 3.5 plotted against the acetic acid concentration used. Fold of induction represents the ratio between the maximum normalized FI at a given acetic acid concentration compared to the maximum normalized basal fluorescence at 0 mM acetic acid. Data obtained from three biological replicates; whiskers show the standard deviation

A construct encoding the improved version of the acetic acid biosensor and the intracellular pH biosensor sfpHluorin (pMM4_14L) was integrated into a pooled CRISPRi strain library [17]. To confirm the function of the biosensor in the new strain background, RFP expression and growth of the pooled CRISPRi Biosensor Library (CBL), were monitored at different acetic acid concentrations, at pH 3.5 (Fig. 2; Additional file 1: Fig. S2). CBL cells cultivated at 10–50 mM acetic acid displayed an increase in reporter signal when exposed to increasing concentrations of acetic acid (Fig. 2). The reporter signal was saturated at around 50 mM of acetic acid as the reporter signal was similar in higher acetic acid concentrations tested. At increasing concentrations of acetic acid, cells had prolonged lag phases and delayed reporter expression (Additional file 1: Fig. S2). For each condition, the peak of the reporter expression was observed during mid-exponential growth of the CBL.

### Screening for strains with enhanced expression of the acetic acid biosensor reporter

**Fig. 3:**
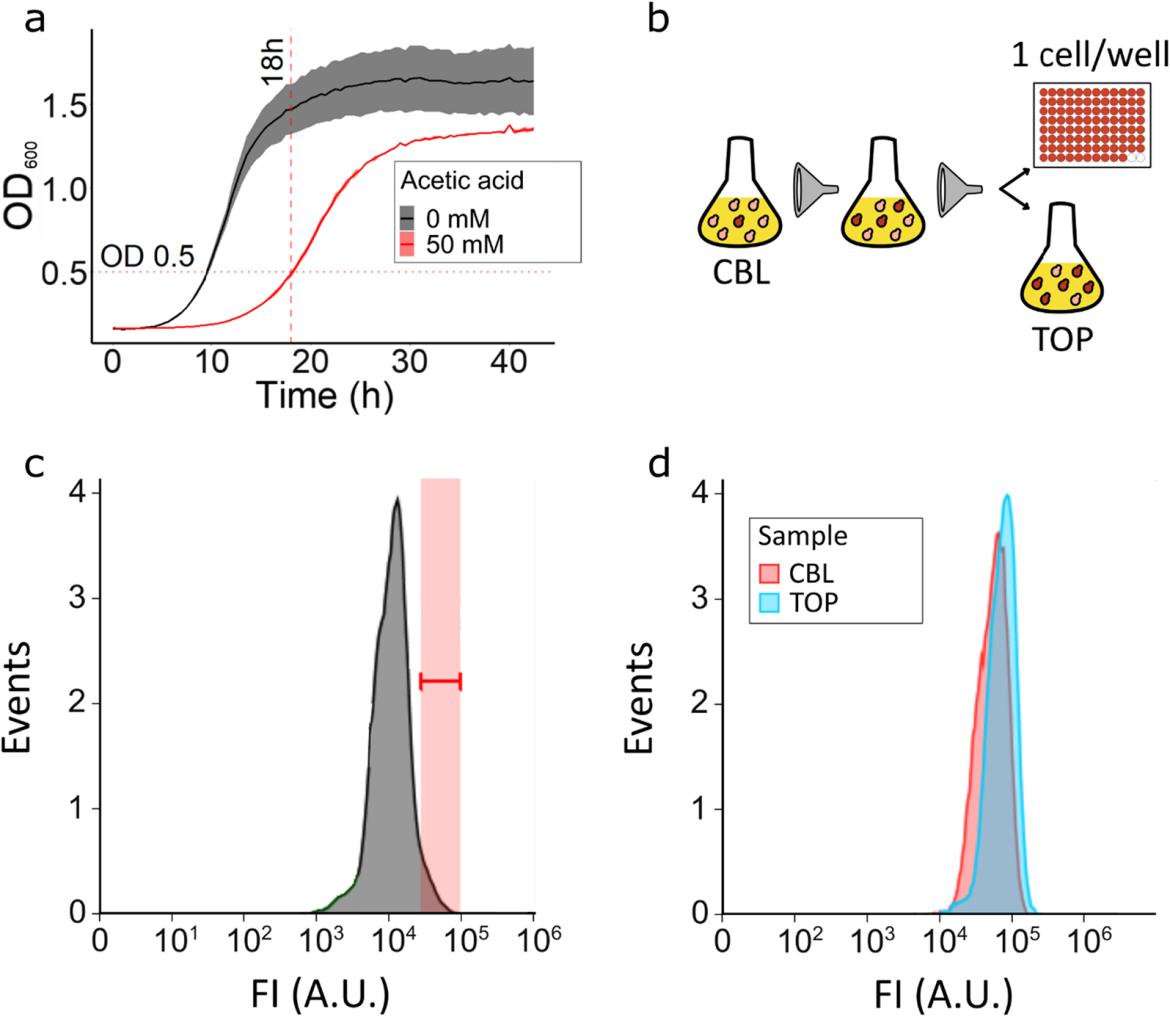
Screening the CRISPRi library through FACS. **a** Growth of the CBL pool at 0 mM (black line) and 50 mM (red line) acetic acid. Time and OD_600_ value at the sampling for FACS are annotated with red dotted lines. Data obtained from two biological replicates; shadowed regions show the standard deviation. **b** The most fluorescent cells were selected by FACS, resulting in 94 isolates and a pooled culture of 50,000 cells (TOP pool). **c** The distribution of fluorescence of the CBL cells at the initial FACS. The gate set for the sorting is represented by the red area. **d** The distribution of fluorescence of the pooled CBL cells (red area) compared to the distribution of fluorescence of cells selected through two rounds of FACS (blue area)

**Fig. 4:**
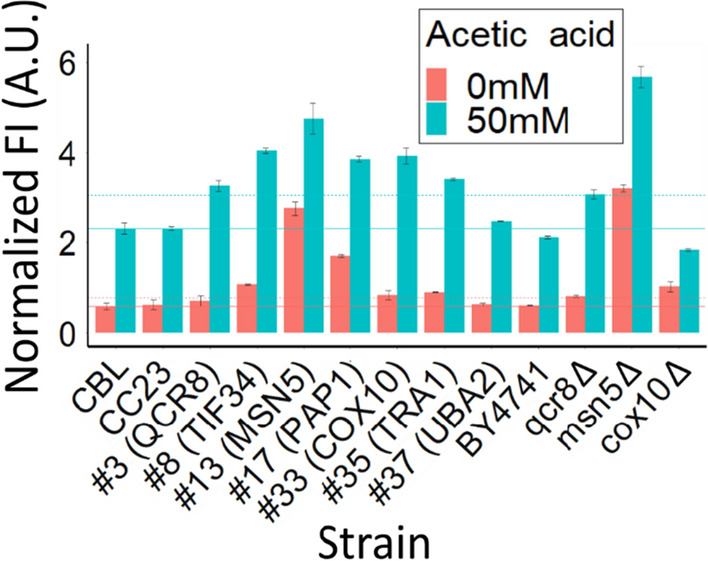
Normalized maximal fluorescence intensity (FI) of the CBL, selected library strains and corresponding available deletion mutants and the parental strain BY4741 at 0 and 50 mM acetic acid, at pH 3.5. FI values present the highest values measured for each strain. The solid lines mark the average value of the CBL, the dashed lines mark a 33% increase in FI compared to the CBL. Data obtained from seven (CBL), three (deletion mutants) or five (other samples) biological replicates; whiskers show the standard deviation

The growth of the pooled CBL library was monitored at 0 and 50 mM acetic acid (Fig. 3a). The CBL pool started growing after approx. 4 or 12 h at 0 and 50 mM acetic acid, respectively. The maximal growth rate of the pooled strains was 0.228 h^−1^ and 0.135 h^−1^ at 0 and 50 mM acetic acid, respectively. The CBL pool was sampled at the middle of the exponential phase, after approx. 18 h of cultivation in acetic acid containing medium (Fig. 3a) and sorted by FACS to enrich the population for cells displaying the highest RFP signal (Fig. 3b–d). After the initial FACS, sorted cells were recovered in liquid media and allowed to grow until stationary phase. These cells were then used to inoculate a fresh preculture that was used for a second FACS. A total of 41 isolates and approx. 50,000 pooled cells (for short, the TOP pool) with the highest RFP signal were analyzed (Fig. 4; Additional file 1: Fig. S3).

At 50 mM acetic acid and pH 3.5, 8 isolated strains displayed a reporter signal 33% higher than CBL (Additional file 1: Figs. S3 and S5). Six of these strains, and 6 other strains with a reporter signal similar or lower compared to the reporter signal of the CBL were characterized and identified through sequencing (Fig. 4; Additional file 1: Fig. S4; Table 1). The biosensor cassette was then also integrated into the *qcr8*Δ, *msn5*Δ, *cox10*Δ mutants of the EUROSCARF deletion collection, and these strains were analyzed for biosensor response at 50 mM acetic acid, pH 3.5 (Fig. 4). In line with what was seen for #8 and #13 with gRNAs targeting *QCR8* and *MSN5*, the *qcr8*Δ and *msn5*Δ strains displayed a reporter signal 33% higher than CBL (Fig. 4). The reporter signal of *msn5*Δ was the highest among all the strains tested (Additional file 1: Fig. S4). The reporter signal of the *cox10*Δ mutant was higher than that of CBL. At 0 mM acetic acid the reporter expression of strains *qcr8*Δ, #8, #13, *msn5*Δ, #17, #33, *cox10*Δ and #35 was 33% higher compared to the pooled CBL library, whereas the other 7 strains displayed a similar fluorescent signal (Fig. 4 and Additional file 1: Fig. S4).

**Table 1:** Properties of isolated strains

Strain	RFP expression*	Growth**	Target gene	Description of target gene	E or RE***
#3	+	ns	*QCR8*	Subunit of the ubiquinol cytochrome-c reductase [40]	RE
#8	+	–	*TIF34*	Subunit of the eukaryotic translation initiation factor 3 (eIF3) [41]	E
#13	+	–	*MSN5*	Karyopherin involved in nuclear import and export of proteins, involved in nuclear export of Haa1 [37, 42]	RE
#15	=	ND	*NDC1*	Subunit of the transmembrane ring of the nuclear pore complex (NPC) [43]	E
#17	+	–	*PAP1*	Poly(A) polymerase [44]	E
#32	=		*CBP2*	Nuclear-encoded mitochondrial protein that binds to RNA to promote splicing [45]	RE
#33	+	–	*COX10*	Heme A farnesyltransferase [46]	RE
#35	+	–	*TRA1*	Subunit of Spt–Ada–Gcn5 acetyltransferase (SAGA) and NuA4 histone acetyltransferase complexes [47]	E
#37	=	ns	*UBA2*	Subunit of heterodimeric nuclear small ubiquitin-like modifier protein (SUMO) [48]	E
#43	=	ND	*RPS30B*	Protein component of the small (40S) ribosomal subunit [49]	E
#46	=	ND	*HSH49*	U2-small nuclear ribonucleoprotein (snRNP) associated splicing factor [50]	E
#49	=	ND	*LCB1*	Component of serine palmitoyltransferase [51]	E

*Reporter expression 30% higher (+) or similar (=) compared to the CBL

**Growth significantly (p < 0.05) decreased (-) or not significantly different (ns) compared to the CC23 control strain. ND = not determined

***Essential (E) and respiratory growth essential (RE) genes are indicated

### Intracellular acetic acid concentration and reporter signal

**Fig. 5:**
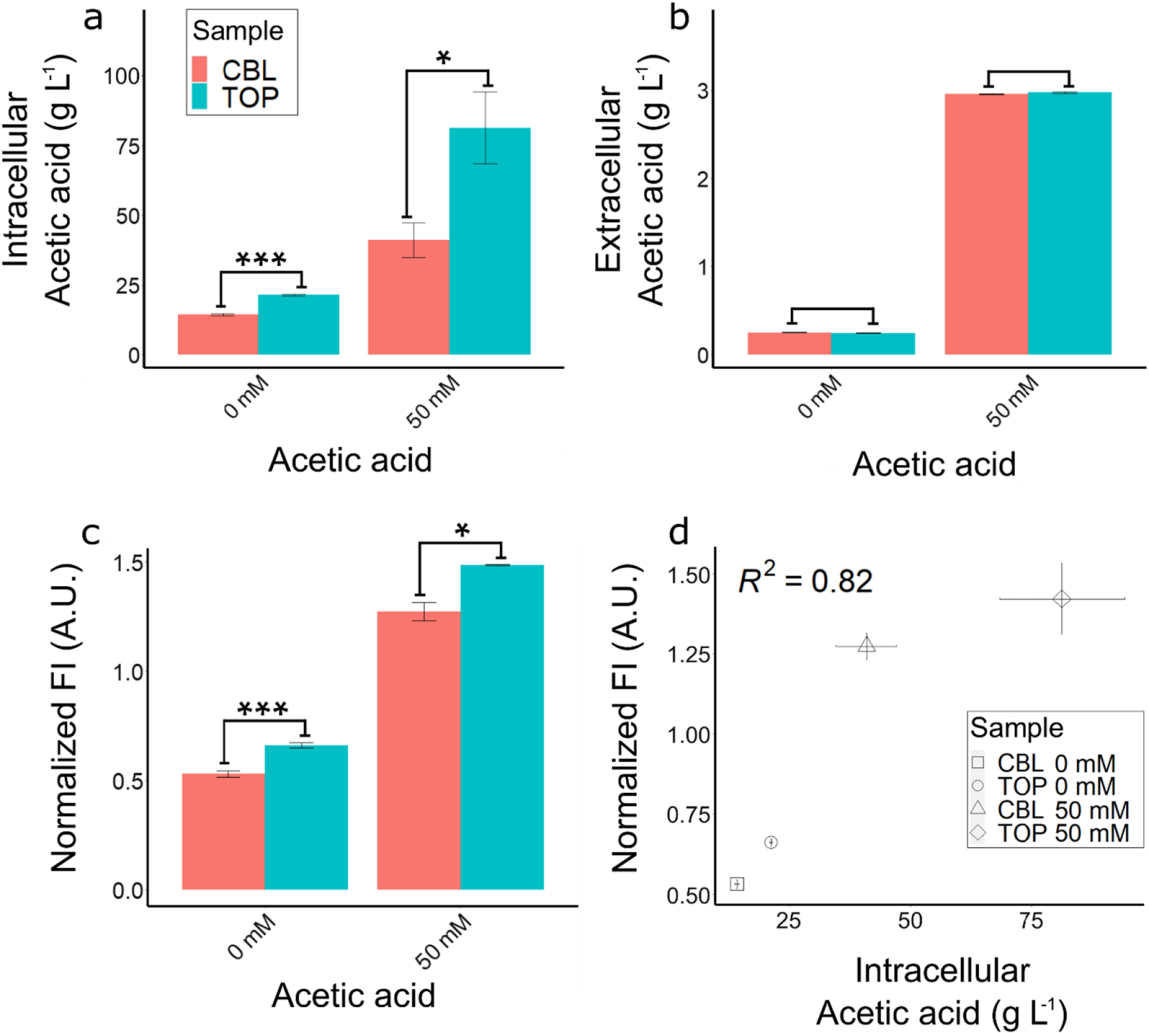
**a** Intracellular and **b** extracellular acetic acid concentrations. **c** Normalized fluorescence intensity (FI) of the CBL and TOP cultures, grown at 0 and 50 mM acetic acid at pH 3.5. **d** Scatterplot displaying the intracellular acetic acid concentrations against the normalized FI of the cultures. The Pearson correlation coefficient (R^2^) is indicated in the plot. Data obtained from three biological replicates; whiskers show the standard deviation. Statistical significance was calculated using the two-tailed two-sample unpaired t-test and is represented as: *p ≤ 0.05; ***p ≤ 0.001

The pooled cells with highest reporter fluorescence (TOP cells) were cultivated at 0 and 50 mM acetic acid and the extra- and intracellular acetic acid concentration, as well as biosensor reporter expression, were measured from samples taken during the logarithmic growth of the cultures (Fig. 5).

The intracellular acetic acid concentration measured from the TOP cells was significantly higher compared to the CBL pool cells, both at 0 (p < 0.001) and 50 mM (p < 0.05) acetic acid (Fig. 5a). The extracellular acetic acid concentration was similar (p > 0.05) for both cultures at the different acetic acid concentrations (Fig. 5b). The reporter signal measured from the TOP cells cultivated at 0 or 50 mM acetic acid was higher than what was measured from the CBL cells at the same condition (p < 0.05) (Fig. 5c). The biosensor reporter fluorescence showed a high correlation (R^2^ = 0.82) to the intracellular acetic acid concentration of the cells (Fig. 5d).

### Biosensor response and intracellular pH and of selected strains

**Fig. 6:**
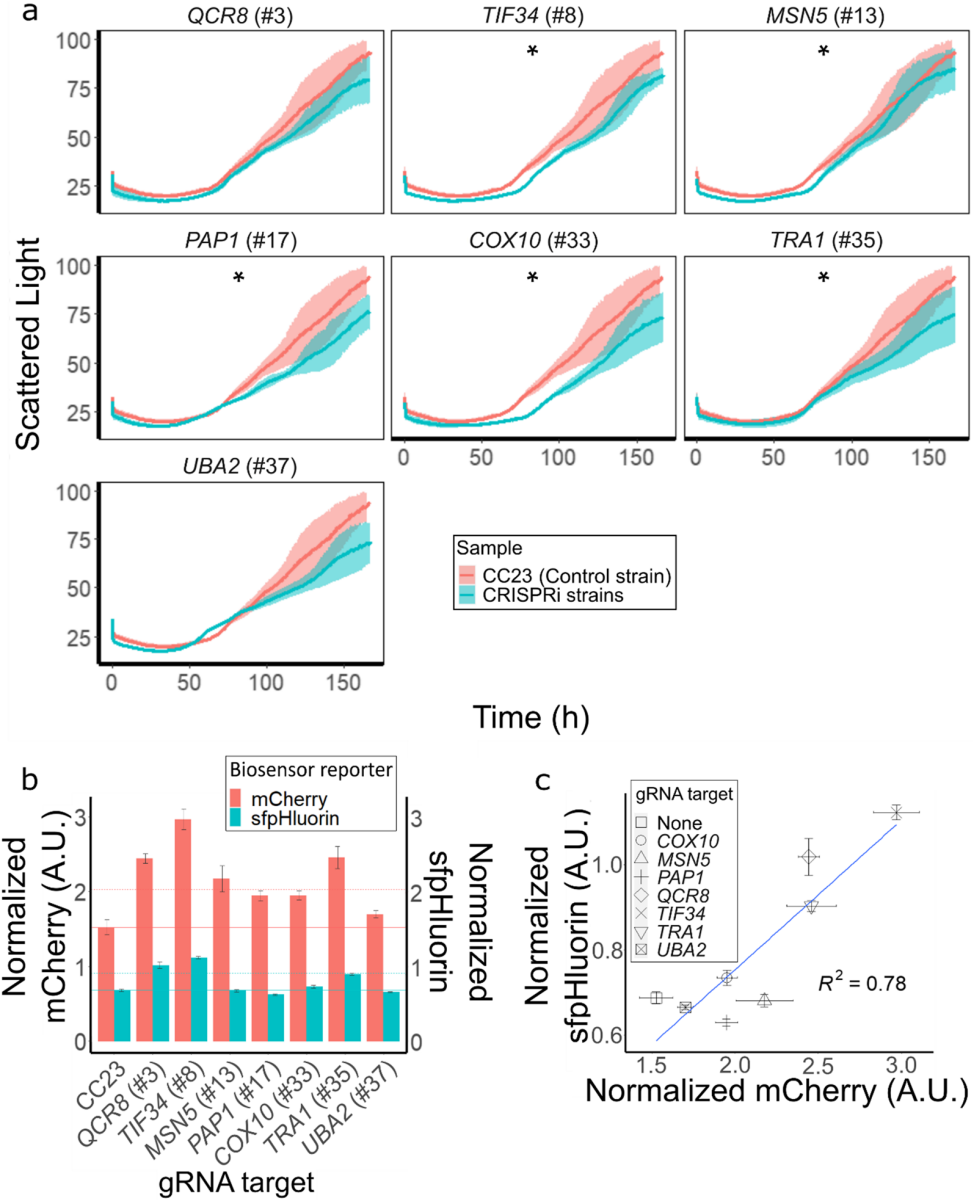
Characterization of selected isolates at 150 mM acetic acid, pH 4.5. **a** Growth of control strain CC23 (red lines) and strains isolated from the CRISPRi library (green lines) measured as scattered light and **b** maximum fluorescence intensity (FI) of the biosensor normalized by biomass (red bars) and sfpHluorin ratio at the time of maximum biosensor output (green bars). The solid lines mark the average normalized FI or sfpHluorin ratio value of the CC23 control strain, the dashed lines mark a 33% increase in normalized FI or sfpHluorin ratio compared to CC23. **c** Maximum fluorescence intensity (FI) of the biosensor plotted against the sfpHluorin ratio of strains at the time of maximum biosensor output. Data obtained from six (CC23) or three biological replicates; whiskers and shadows show the standard deviation Statistical significance among growth curves was calculated using the “compareGrowthCurves” permutation test in R [52]; *p < 0.05

Seven strains selected by FACS as well as the CC23 control strain were grown at 150 mM acetic acid, pH 4.5, where the lag phase was 30–50 h (Fig. 6a) for all strains. At 150 mM strains #8, #13, #17, #33 and #35 with gRNAs targeting *TIF34*, *MSN5, PAP1, COX10* or *TRA1* had a significantly impaired growth (p < 0.05) compared to the control strain, CC23. Isolates #3, #8, #13 and #35 with gRNAs targeting *QCR8, TIF34*, *MSN5* or *TRA1* displayed a >30% higher maximum FI compared to the control strain CC23 (Fig. 6b). Notably, strains #3, #8 and #35 with gRNAs targeting *QRC8, TIF34* or *TRA1* showed >60% increase in maximum FI.

SfpHluorin was used to monitor the intracellular pH of selected strains. Strains with gRNAs targeting *QCR8, TIF34* and *TRA1 (*#3, #8, #35) showed a ≥33% increase in the sfpHluorin ratio when compared to the CC23 control (Fig. 6b). A higher sfpHluorin ratio indicates a higher cytosolic pH [24, 39]. The comparison between the maximum reporter expression of the acetic acid biosensor observed for each strain and the respective sfpHluorin ratio calculated at the time of maximum biosensor output, displayed a strong positive correlation (R^2^ = 0.78, Fig. 6c).

## Discussion

In large strain libraries, only a very limited subset of variants is expected to show improved performance observes [17, 18]. Efficient ways to screen and select for specific phenotypes are therefore of great value. In this study, we exploited an acetic acid biosensor [22] to screen a CRISPRi strain library covering all essential and respiratory growth essential genes [17], and to identify genes involved in acetic acid sensitivity. The acetic acid biosensor was used to select cells with a higher intracellular acetic acid content (Fig. 5).

One of the most important features of a biosensor is its dynamic range. One way to improve the dynamic range of biosensors is changing or modifying the promoter that drives the expression of the TF of the biosensor [19]. Following this strategy, biosensors responsive to, e.g., naringenin or cis,cis-muconic acid [53] or fatty acyl-CoA [54] have been developed. In those studies, the TFs of the biosensor were expressed under the control of different promoters to determine the best dynamic range. The weak *REV1* promoter was optimal for driving the TF of the cis,cis-muconic acid biosensor [53], whereas strong promoters such as p*TDH3* [53] and p*TEF1* [54] were suited for expression of TFs in the naringenin [53] or fatty acyl-CoA biosensor [54]. In our study, we obtained a 20% higher dynamic range compared to the original biosensor when the native *HAA1* promoter driving the sTF was replaced by the medium-weak constitutive promoter of *RET2* (Fig. 1; Additional file 1: Fig. S1). Stronger promoters like p*TDH3* (in yMM4_3) or p*TEF1* (yMM4_7), as well as weaker promoters like p*PSP2* (yMM4_20) or p*POP6* (yMM4_18), resulted in a faster reporter saturation even at 0 mM acetic acid, or in poor expression of the reporter.

In our earlier study [22], we determined that the biosensor did not induce expression of a fluorescent gene under the control of the Haa1-regulated promoter *YGP1*, thus we concluded that it is unlikely that the biosensor shows any major interference with the endogenous Haa1 regulatory network. Still, an additional motivation for replacing the *HAA1* promoter was to avoid any interplay with native regulation of Haa1. Haa1 is a crucial TF for the cellular response to weak acid stress and is known to be up-regulated under a range of stress conditions [55, 56]. In earlier studies, the expression of *RET2* was not reported to be altered in response to acetic acid [57–59].

The operational range of the new acetic acid biosensor was consistent with that of the original biosensor, spanning from 10 to 60 mM acetic acid (0.6–3.6 g L^–1^; Fig. 2). The operational range (reporting 0-50 mM acetic acid, Fig. 2b) of the biosensor was in the CRISPRi strains similar to what was seen when the biosensor was expressed in CEN.PK113-5D [22]. The dynamic range of the biosensor when expressed in the CRISPRi strains was however narrower, reaching approx. 3.5-fold of induction, versus a 6-fold induction measured in CEN.PK113-5D [22]. Some differences in biosensor performance when the host was changed have been seen for other biosensors [53, 60, 61]. Nevertheless, biosensors with similar or even smaller dynamic ranges have successfully been used for FACS-based screenings [20, 21]. Dabirian et al. [20] successfully screened an overexpression library for genes enhancing fatty acyl-CoA pools using a biosensor with a dynamic range reaching 3.3-fold of induction. The number of strains of the biosensor library in this study [20] was approx. 6 times that of the original MoBY-ORF strain library, whereas Wang *et al*. [62] successfully screened a naringenin biosynthetic library at a coverage of approx. 3 times, thus the 11x coverage of the CBL in this study should be plenty.

A common way to screen pooled CRISPRi libraries in *S. cerevisiae* is through competitive growth assays, where strains with a phenotype that gives a growth-advantage under a set condition outcompete those with no change in phenotype or decreased fitness [13, 15–17]. This setup favors cells with shorter lag phase and this advantage is amplified over time due to the progressive reduction of nutrients available [18]. Subsequentially, cells with delayed growth will be under-represented in the final analysis. In our study, we sorted cells when the pooled cell population displayed highest fluorescence, after only a few doublings. Then, we selected strains based on the single-cell specific reporter expression, without any evident bias for cells with shorter lag phases. This way, we could isolate cells with higher reporter signal and decreased tolerance to acetic acid (strains #8, #13, #17, #33 and #35) compared to the control strain (Fig. 4; Fig.6). As previously observed [17, 18], all strains grew similarly at 0 mM acetic acid (Additional file 1: Figs. S3, S4). At 50 mM acetic acid, the lag phases of the individual strains varied greatly (Additional file 1: Figs. S3, S4); 28 of the 41 isolates had a >1 h shorter lag phase compared to the CBL, suggesting a higher acetic acid tolerance. All isolates except strain #43, with gRNA targeting *RPS30B*, had a shorter lag phase compared to the pooled TOP cells at 50 mM acetic acid. There was a strong correlation (R^2^ = 0.82) between the acetic acid reporter signal and intracellular acetic acid accumulation of the pooled TOP cells and the complete CBL pool (Fig.5). At 50 mM acetic acid, the pooled TOP cells displayed delayed growth compared to the complete CBL pool in medium with 50 mM acetic acid (Additional file 1: Figs. S3, S4). It may be that the higher acetic acid concentration caused the prolonged lag phase. High concentrations of intracellular acids have been reported to be detrimental for the cell [25, 63], especially at low pH [64]. Exposure to acetic acid predominantly leads to increased lag phases, the length of which increase as the acetic acid concentrations increase [18, 63].

Acetic acid tolerance is reported to correlate with the capacity of the cells to extrude protons and thereby maintaining a neutral intracellular pH [65]. We set to monitor the intracellular pH using sfpHluorin and found a strong (R^2^ = 0.78) correlation between the reporter of the acetic acid biosensor (indicating the intracellular acetic acid retention) and the sfpHluorin signal. Unexpectedly, the sfpHluorin ratio of UV-GFP/GFP fluorescence measured at the time when the acetic acid biosensor signal peaked, grew as the acetic acid biosensor signal increased, indicating a higher cytosolic pH [24, 39] in cells with a higher biosensor signal. We speculate that the strains isolated that presumably retained more acetic acid may still have been able to extrude protons and thereby maintain a higher intracellular pH. For this to be possible, the buffering capacity of the cells has to be altered. The sfpHlourin ratios measured from the cells varied from 0.63 to 1.12, representing an intracellular pH of 4.8 to 5.9. Similar intracellular pH values upon acetic acid have been reported earlier; Dong *et al.* [58] determined the intracellular pH of cells at 150 mM acetic acid (at pH 3.0) to be approx. 4 whereas Torello Pianale et al. [24] measured the intracellular pH to be approx. 6 in cells at 75 or 100 mM acetic acid (at pH 5.0). The pKA of acetic acid is 4.76, thus a lower intracellular pH at lower medium pH is plausible.

Six of the isolated strains and the *qcr8*Δ and *msn5*Δ mutants of the EUROSCARF deletion collection displayed a > 33% increased fluorescence compared to the CBL pool at 50 mM acetic acid (Fig. 4). These strains contain gRNAs targeting genes coding for proteins with diverse functions (Table 1). This may be expected, as previous work has revealed a great number of genes to be involved in acetic acid tolerance [5]. Deletion of genes often increases the acetic acid sensitivity [5] and similarly strains #8, #13, #17, #33 and #35, with gRNAs targeting *TIF34*, *MSN5, PAP1, COX10* or *TRA1* showed significantly (p > 0.05) increased acetic acid sensitivity (Fig. 6). *TIF34* and *PAP1* are essential genes with different functions, both involved in gene expression (Table 1) [41, 44]. The expression of *TIF34* has previously been reported to be upregulated in cells exposed to acetic acid [66], and a strain with a gRNA targeting *TIF34* (encoding a subunit of the eIF3 core complex) was identified to have increased acetic acid sensitivity in a previous screening of the CRISPRi library [18]. Similarly, a strain with a gRNA targeting *SUI1* (encoding another translation initiation factor which directly interacts with eIF3 and eIF5 [87, 88]) displayed a higher sensitivity to acetic acid [18]. Moreover, Cheng *et al*. [86] has showed that another initiation factor (eIF5A) plays part in acetic acid tolerance regulation. They demonstrated that the eIF5A-Ume6 switch regulates tolerance to acetic acid in several ways. Isolation of eIF3 revealed that the core of the complex (composed by the five subunits Tif32, Prt1, Nip1, Tif35 and Tif34) was associated with eIF5 [89]. Thus, it is possible that the eIF3 complex that Tif34 is a part of influences acetic acid tolerance in yeast. Therefore, the downregulation of *TIF34* may result in misregulation and increased acetic acid sensitivity.

*PAP1* has, to our knowledge, not been mentioned earlier in studies concerning acetic acid tolerance. Nonetheless, polyadenylation by *PAP1* could increase the stability of the transcript of genes related to acetic acid resistance. Thus, a fully functional transcriptional system may be crucial in ensuring mRNA maturation of all components leading to acetic acid tolerance and more studies are needed to elucidate the role of Pap1 in acetic acid tolerance.

The deletion of the respiratory chain gene *COX10*, has previously been shown to result in increased acetic acid sensitivity [67]. *COX10* encodes a heme A farnesyltransferase, catalyzing the first step of the protoheme conversion to heme A prosthetic group [46]. Heme A is required for cytochrome c oxidase activity, which is a central mitochondrial respiratory chain component catalyzing the transfer of electrons from reduced cytochrome c to molecular oxygen [68]. The connection between mitochondrial disfunction and acetic acid sensitivity was highlighted by Sousa et al. [69]*,* even though in this study the tolerance to acetic acid was increased in *cox10*Δ mutants cultivated at high acetic acid concentration (400 mM). Thus, it appears that *COX10* is involved in response to acetic acid stress in a condition-dependent manner. In line with this, when the biosensor was expressed in the *cox10*Δ mutant, the reporter signal was lowered compared to CBL, even though the reporter signal of #33 with gRNA targeting *COX10* was increased (Fig. 4). Earlier screens of the deletion collection [67, 69] or the CRISPRi collection [18] also highlighted that while some of the COX mutants are found among sensitive strains, others are found among the most acetic acid tolerant strains.

In line with our study, the screen by Mukherjee et al. [18], identified a strain expressing a gRNA targeting *MSN5* to grow slower compared to the control. Strain #13 that had a higher biosensor signal and a somewhat reduced growth at 150 mM acetic acid, has a gRNA targeting *MSN5*. *MSN5* encodes a nuclear exportin involved in the relocation and regulation of several TFs [37, 70, 71]. Deletion of *MSN5* has been reported to cause constitutive nuclear localization of phosphorylated TFs, such as Haa1 [37, 70, 72]. Haa1 regulates a network of genes involved in acetic acid stress responses [73] and the binding of acetate allows Haa1 to bind to DNA [37]. Deletion of *MSN5* was shown to lead to reduced levels of Msn2 [74], another TF that has been shown to be important for tolerance to acetic acid [56]. The nuclear localization of Haa1 has been shown to contribute to its destabilization [37]. Therefore, it is plausible that *MSN5* repression may lead to an increased nuclear localization of TFs important to acetic acid stress. Still, the repression of *MSN5* may also contribute to destabilization of TFs, leading to a lower activity and thus to an impaired stress response. The lower biosensor reporter induction of the strain with gRNA targeting *MSN5* (approx. 1.7-fold) compared to the CBL pool (approx. 3.7-fold) when cultivated at 0 and 50 mM acetic acid (Fig. 4), supports this observation. Still, it is important to note that Haa1 is the core component of the acetic acid biosensor that relies on the capacity of Haa1 to bind acetate ions and relocate into the nucleus [22]. Cells where *MSN5* is downregulated may therefore retain more of the sTF of the acetic acid biosensor (BM3R1-Haa1-mTurquoise2) in the nucleus *per se*, leading to higher expression of the biosensor due to more likely binding of BM3R1 to its binding sites in the *ENO1* core promoter driving the reporter expression. The observation that the reporter expression of the strain with gRNA targeting *MSN5* as well as in the *msn5Δ* mutant was significantly higher compared to that of the CBL pool (Fig. 4) supports this observation.

*TRA1* encodes a subunit of SAGA and NuA4 histone acetyltransferase complexes that is reported to interact with TFs, leadings to transcription activation [47]. *TRA1* has been altered through targeted mutagenesis, leading to mutants with increased temperature sensitivity and reduced growth in various media [75]. Interestingly, the mutants had a twofold or greater change in expression of ∼7% of yeast genes in rich media. Notably the expression of *AFT1*, encoding a transcription factor involved in regulation of the oxidative stress response [76], *CWP2*, encoding a major constituent of the cell wall involved in low pH resistance [77] and *PDR12*, encoding a transporter protein known to be important for acetic acid tolerance [63] where among the upregulated genes in the *TRA1* mutants [75]. Other components of the SAGA and Nu4A complexes have previously been reported to be involved in acetic acid tolerance [58]. Overexpression or deletion of *ADA2*, *SGF29* and *YAF9,* genes involved in histone acetylation/deacetylation, was shown to result in enhanced cell death upon acetic acid stress [58]. Several SAGA component mutants of the EUROSCARF collection (*NGG1*, *SPT3*, *SPT7*, *SPT8*, *SPT20* [67, 69]) have been reported to display a compromised growth in acetic acid media. Moreover, repression of *ADA2* and *TAF12* of the SAGA complex or *EPL1* of the NuA4 complex (*EPL1*) was shown to lead to acetic acid sensitivity [18]. Thus, it may that acetylation imbalance plays a role in acetic acid sensitivity. Fine-tuning the expression of *TRA1* may be a way to tune many of the cellular responses towards acetic acid and other stresses, leading to more robust and efficient strains for the production of biobased chemicals.

## Conclusions

To conclude, we have demonstrated that our acetic acid biosensor was able to report intracellular acetic acid retention. We successfully applied the biosensor to screen strains of a CRISPRi library using FACS and isolated five strains with higher acetic acid sensitivity. With the exception of *PAP1*, the other genes repressed in the isolated strains were previously found to be involved in sensitivity to acetic acid or stress conditions associated with acetic acid stress. Fine-tuning the expression of the genes targeted in the acetic acid sensitive strains, *TIF34*, *PAP1*, *TRA1* and *COX10*, may lead to strains with improved acetic acid tolerance. In summary, we have showed that our acetic acid biosensor is a valuable tool for high-throughput screens of mutants relevant for acetic acid tolerance. The acetic acid biosensor could also be used to guide metabolic engineering and to monitor the performance of strains engineered for increased acetic acid tolerance, thus serving as a tool for cell factory development.

## Methods

### Yeast strains and CRISPRi library

For construction of the biosensor variants, *S. cerevisiae* CEN.PK113-5D [78] was used as the parental strain. The CRISPRi strain library (derived from BY4742) screened originally contained 9,078 strains, where 1117 essential genes and 514 respiratory growth essential genes in *S. cerevisiae* (representing over the 98% of both groups) are targeted by 1-16 gRNAs each [17]. The expression of the gRNAs is regulated by a tetracycline-regulatable repressor, controlling a modified Pol III promoter. The gRNAs are thus only expressed in presence of the inducing agent, anhydrotetracycline (ATc). The dCas9-Mxi1 construct of the CRISPRi strains, instead, is expressed constitutively under the strong *TEF1* promoter. The *cox10*Δ, *msn5*Δ and *qcr8*Δ mutants and the BY4741 parental strain were taken from the EUROSCARF collection [90].

### Design of constructs and modular cloning

All genetic constructs were based on the acetic acid-binding TF Haa1 and the bacterial repressor BM3R1 [22] and cloned following the MoClo method [79]. In the construction of the new biosensors for improving the dynamic range, the level-0 plasmid pMM0_1 harboring the *HAA1* promoter that was used in the original biosensor, was replaced by a series of other vectors containing different promoters (pLT03, pLT0_9 and pYTK009 to pYTK026; Additional file 2: Table S1). The resulting level-1 plasmids, expressing the sTF open reading frame (ORF) *BM3R1-HAA1-mTurquoise2* under different promoters, were named from pMM1_18 to pMM1_37. In plasmid pMM1_46 *sfpHluorin* [39] was expressed under the *TDH3* promoter as described earlier [24]. All plasmids and primers used in the study are listed in Additional file 2. Plasmids used in the study are shared through Addgene.

Level-2 vectors, harboring the new versions of the acetic acid biosensor, were assembled by combining plasmids pMM1_1, pMM1_18 to pMM1_37 and pMM1_8. The resulting integration plasmids (pMM4_1 to pMM4_20) harbored homology arms for the *HO locus*, the *URA3* marker for selection in yeast and the sTF encoding construct *BM3R1-HAA1-mTurquoise2* under different promoters and *mCherry* under the *ENO1* core promoter including binding sites for BM3R1. The final construct integrated into the CRISPRi library strains pMM4_14L, was assembled by combining pMM1_1f, pMM1_31, pMM1_8c and pMM1_46). A set of point mutations were observed in the mCherry ORF of the pMM4_14L

plasmid integrated: N28D, N97K, C143K, R144K, T152S, D201N, and L207T. *Escherichia coli* DH5α cells were used for plasmid construction, primers were purchased from Eurofins Genomics, and PCR components from Thermo Scientific. Plasmids were purified using the GeneJET Plasmid Miniprep Kit (Thermo Scientific).

### Integration of biosensors

The different variants of the biosensor (pMM4_1 to pMM4_20) were digested with NotI and transformed into CEN.PK113-5D using the Gietz method [80]. The biosensor with the sTF encoding construct *BM3R1-HAA1-mTurquoise2* expressed under the *RET2* promoter together with the sfpHluorin expression cassette (pMM4_14L) was integrated into the *HO locus* of the CRISPRi library strains [17] and to the control strain (CC23) harboring a gRNA that is non-homologous to the *S. cerevisiae* genome, as well as to the *cox10*Δ, *msn5*Δ, *qcr8*Δ mutants and BY4741. Expression under the *RET2* promoter was previously described to be constitutive and rather weak [79]. The library strains were transformed with 5 µg of NotI digested pMM4_14L following the method described by Benatuil *et al.* [81]*.* Cells were electroporated at 1.5 kV, in a BioRad GenePulser cuvette (0.2 cm electrode gap) and plated on solid YPD medium containing zeocin (YPD, 100 µg mL^-1^ zeocin, 20 g L^-1^ agar-agar) at 30°C for 3-5 days. A total of 15 transformations were performed.

After the transformations, the resulting cell forming units (CFUs) were counted and construct integration was verified by colony PCR. Approx. 10,000 CFUs were obtained per µg of DNA, resulting in a theoretical coverage of 11-fold the CRISPRi library. The transformation series led to approx. 100,000 CFUs and the biosensor library was named the CRISPRi Biosensor Library, for short CBL. A pooled library was created by scraping the cells from the plates and re-suspending into YPD glycerol stock solutions (YPD, 17% glycerol (v/v)). The pooled library was aliquoted and stored at -80°C.

### Cultivation and screening conditions

Unless otherwise specified, yeast cells were cultivated in synthetic complete medium (SC) (0.77 g L^−1^ complete supplement mix drop out (CSM), 6.9 g L^−1^ yeast nitrogen base without amino acids (YNB w/o AA), 20 g L^−1^ glucose, pH 5.5, 4.5 or 3.5) containing 2 µg mL^−1^ ATc (SC-ATc), in white 96-well plates (Greiner CELLSTAR®, Sigma-Aldrich) with 200 µL medium, in 24-well plates (Enzyscreen) with 2.5 mL medium or in 100 mL shake flasks with 20 mL medium. All cultures were inoculated to an OD_600_ of 0.1 from a preculture (5 mL, grown for 48 h in SC media at pH 4.5 for cultures at pH 4.5 or at pH 5.5 for cultures at pH 3.5). All precultures were inoculated with approx. 9 x 10^6^ cells from respective cryostock. Acetic acid, as well as ATc, were added to the media at the beginning of the cultivation. The added acetic acid was diluted into distilled water at a concentration of 500 mM and the pH of the solution was adjusted to the pH of the medium through NaOH titration. The ATc stock solution was prepared by dissolving ATc into DMSO to a concentration of 125 µg mL^−1^. Plates with 200 µL-cultures were cultivated at 30 °C and 85% humidity, shaking at 995 rpm, using a microbioreactor device (Biolector, Beckman Coulter / m2p-labs). Plates with 2.5 mL-cultures (in 24-well plates) were cultivated at 30 °C, shaking at 250 rpm, using a Growth profiler 960 (Enzyscreen). Flask cultures were incubated at 30 °C and 220 rpm in shakers.

For fluorescence-activated cell sorting (FACS), cells were cultivated until stationary phase (approx. 40h) in 24-well plates and re-inoculated for cultivation until mid-exponential phase, corresponding to an OD_600_ of approx. 0.5. Screening of the pooled and single cell cultures sorted by FACS was performed in two biological replicates, in SC medium at 0 and 50 mM acetic acid at pH 3.5. Selected strains were further characterized in three biological replicates at 150mM acetic acid, at pH 4.5. The growth of the cultures was analyzed according to [82]. Growth curves were compared for significant differences using the “compareGrowthCurves” permutation test in the R Stats package (R studio Version 1.2.5019) [52].

### Fluorescence measurements and FACS

Fluorescence intensity (FI) was measured from cultures grown in 96-well plates in the Biolector. Red fluorescence was measured through the mCherry/RFPII filter (filter code E-OP-319, excitation 580 nm, emission 610 nm, gain 100) whereas GFP/sfpHluorin fluorescence was measured through the UV-GFP (filter code E-OP-341, excitation 400 nm, emission 510 nm, gain 20) and GFP filters (filter code E-OP-304, excitation 488 nm, emission 520 nm, gain 40). The signal from sfpHluorin was reported as the ratio between the fluorescent signals measured through the two filters (UV-GFP/GFP). The red fluorescence measured was normalized against the biomass of the cultures, measured as scattered light (excitation 620 nm, emission 620 nm, gain 20). The fold of activation of the acetic acid biosensor was calculated as the ratio between the fluorescence at a given acetic acid concentration and the fluorescence measured in cultures at 0 mM acetic acid. Unless otherwise specified, data presented are the average of three biological replicates.

FACS was performed using a Sony SH800 cell sorter (Sony Biotechnology) with a yellow-green laser (561 nm) detected in the 600/60 nm channel. The data for 100,000 cells were recorded for library strains samples to set up gates for single cells and to enrich for cells with high RFP intensity (top 5%). Sorting was done on purity mode, meaning that a droplet was sorted if it contained one or more targeted events of a single type and the nearest edge region of both of the adjacent droplets was empty or non-conflicting. At least 50,000 cells were sorted. Sorted cells were recovered in 2.5 mL of YPD medium, grown for 48 h at 30 °C and subjected to a second round of sorting with the same gates, mode and number of sorted cells. Additionally, in the second round of sorting, 96 single cells from the top 5% of the fluorescent population were sorted in a 96-well plate, where each well contained 200 µL of YPD. Cells recovered from the second round of sorting were grown for 48 h at 30 °C and stored at −80 °C in YPD glycerol stock solutions. This population of cells is referred to as the TOP population.

### Intracellular acetic acid measurement

High-performance liquid chromatography (HPLC) was used to measure acetic acid and glucose concentrations of culture samples. A 20 mL shake flask culture was harvested after 18 h, at an OD_600_ of approx. 0.5, and samples were centrifuged at 3000 rpm, 4 °C for 10 min. Extraction of intracellular metabolites was done as described using a slightly modified protocol [22], originally developed by Ilmén et al. [83]. In brief, the cell pellet was washed in 20 mL of ice cold 1M Tris-HCl solution at pH 9.0 and resuspended in 10 mL of ice cold 5% (w/v) trichloroacetic acid (TCA) solution. Cells in TCA were vortexed for 1 min, incubated on ice for 30 min, vortexed again for 1 min and centrifuged at 5500 rpm for 30 min at 4 °C. After this, the supernatant was collected, diluted, and filtered before being measured using a Jasco UV-RI HPLC (LC-4000 series) equipped with an AS-4150 auto-sampler, a CO-4061 column, a RI-4031 RI detector and a UV-4075 UV detector. Compounds were separated using 5 mM H_2_SO_4_ at 80 °C, with a flow rate of 0.8 mL min^−1^. The intracellular concentration of acetic acid was normalized against the cell volume. A CASY device (Schärfe System GmbH) was used to assess cell volume and the number of cells in the culture. Ten µL of culture sample were diluted in 10 mL of CASY ton buffer solution (Roche Innovatis) and three measurements were performed on each sample. Data were analyzed using the CromNAV software.

### Identification of strains through sequencing

The genomes of selected enriched strains were extracted using YeaStar Genomic DNA Kit (Zymo Research) and the gRNA region was PCR amplified using primers pair MM68/IL116 (Additional file 2: Table S3) and Phusion High-Fidelity DNA Polymerase (Thermo Fisher Scientific). The PCR products were sequenced by a Macrogen Europe.

## Supplementary Information

**Additional file 1: Figure S1.** Characterization of new biosensor variants. **Figure S2.** Growth and normalized fluorescence intensity of the CRISPRi library cultures expressing the biosensor (CBL) at different concentrations of acetic acid. **Figure S3**. Normalized fluorescence intensity and growth measured as scattered light of isolated strains, the TOP pool and the CBL pooled at 0 and 50 mM acetic acid. **Figure S4.** Normalized maximal FI of the pooled library (CBL) as well as selected strains of the CRISPRi library expressing the biosensor in the presence and absence of 50 mM acetic acid. **Figure S5**: Length of lag phase and time to reach the peak in reporter of the CBL pool the TOP pooled and strains isolated, at 0 and 50 mM acetic acid. **Figure S6**. Growth and normalized fluorescence intensity of selected isolates at 150 mM acetic acid.

**Additional file 2: Table S1. **Level-0 plasmids used in this study. **Table S2.** Level-1 and level-2 plasmids used in this study. **Table S3.** Oligonucleotides used in this study.

## Data Availability

The datasets supporting the conclusions of this article are included in the article and its Additional files.

## Abbreviations

CBL: CRISPRi Biosensor Library
CRISPRi: CRISPR interference
gRNA: Guide RNA
HPLC: High-performance liquid chromatography
OD: Optical density
SC: Synthetic complete
sTF: Synthetic transcription factor
TF: Transcription factor
TOP: Higher fluorescence cell pool
