# Supplementary material for: Identification of acetic acid sensitive strains through biosensor-based screening of a Saccharomyces cerevisiae CRISPRi library
Source: Microb Cell Fact. 2022 Oct 15;21:214. doi: 10.1186/s12934-022-01938-7 (PMC9571444; doi:10.1186/s12934-022-01938-7)
Supplement: Supplementary file 1 — Additional file 1: Figure S1. Characterization of new biosensor variants. Figure S2. Growth and normalized fluorescence intensity of the CRISPRi library cultures expressing the biosensor (CBL) at different concentrations of acetic acid. Figure S3. Normalized fluorescence intensity and growth measured as scattered light of isolated strains, the TOP pool and the CBL pooled at 0 and 50 mM acetic acid. Figure S4. Normalized maximal FI of the pooled library (CBL) as well as selected strains of the CRISPRi library expressing the biosensor in the presence and absence of 50 mM acetic acid. Figure S5: Length of lag phase and time to reach the peak in reporter of the CBL pool the TOP pooled and strains isolated, at 0 and 50 mM acetic acid. Figure S6. Growth and normalized fluorescence intensity of selected isolates at 150 mM acetic acid. [file 12934_2022_1938_MOESM1_ESM.docx]

**Identification of acetic acid sensitive strains through biosensor-based screening of a *Saccharomyces cerevisiae* CRISPRi library**

**Maurizio Mormino, Ibai Lenitz, Verena Siewers, Yvonne Nygård**

**Additional file 1**

**Figure S1.** Characterization of new biosensor variants.

**Figure S2.** Growth and normalized fluorescence intensity of the CRISPRi library cultures expressing the biosensor (CBL) at different concentrations of acetic acid.

**Figure S3**. Normalized fluorescence intensity and growth measured as scattered light of isolated strains, the TOP pool and the CBL pooled at 0 and 50 mM acetic acid.

**Figure S4.** Normalized maximal FI of the pooled library (CBL) as well as selected strains of the CRISPRi library expressing the biosensor in the presence and absence of 50 mM acetic acid.

**Figure S5**: Length of lag phase and time to reach the peak in reporter of the CBL pool the TOP pooled and strains isolated, at 0 and 50 mM acetic acid.

**Figure S6**. Growth and normalized fluorescence intensity of selected isolates at 150 mM acetic acid.


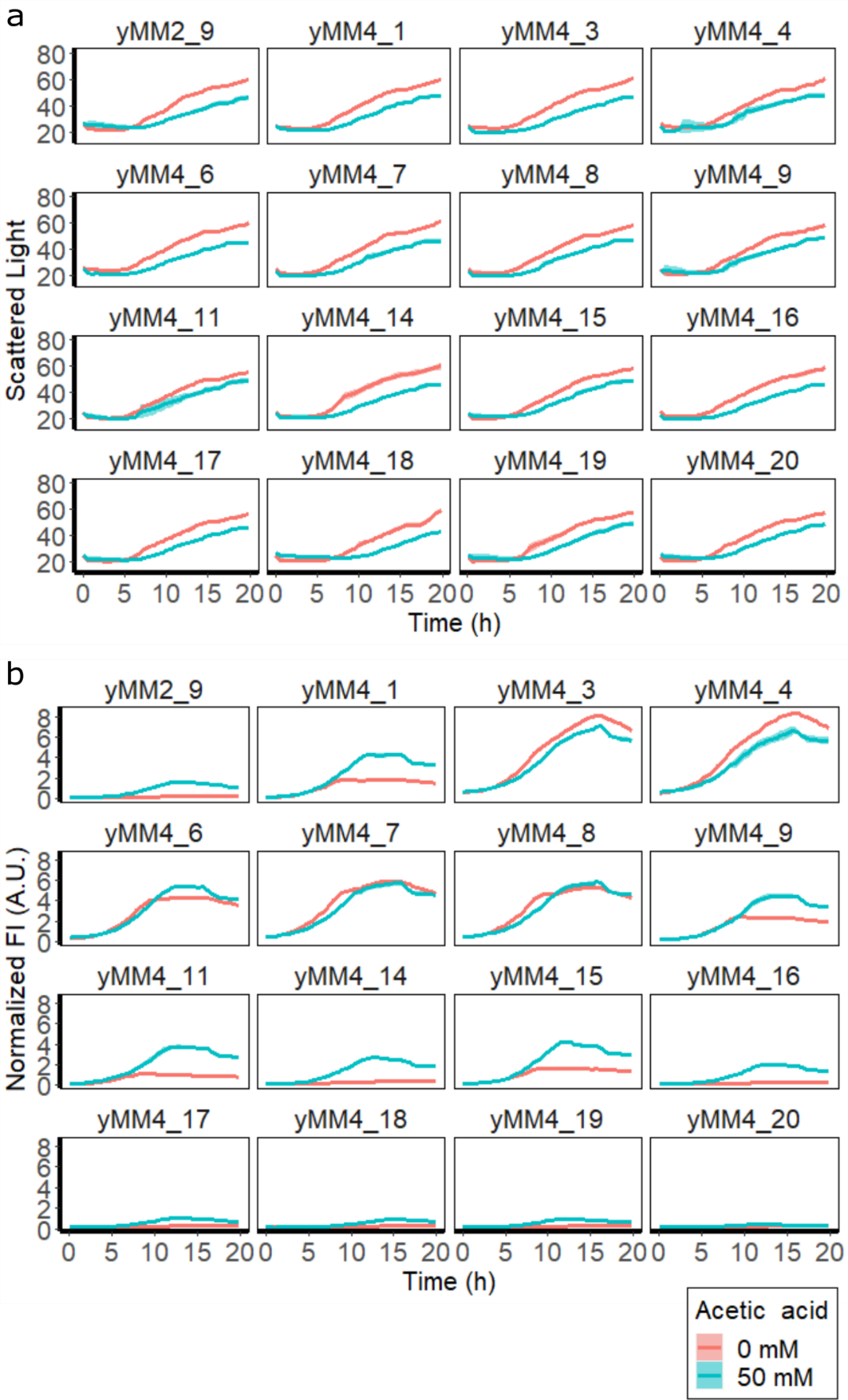


**Figure S1.**  Characterization of new biosensor variants. a) Growth and b) normalized fluorescence intensity (FI) of the original biosensor strain (yMM2_9) and of strains with new versions of the acetic acid biosensor at 0 (cyan lines) and 50 mM (red lines) acetic acid. The strains were grown in microbioreactors. Data obtained from three biological replicates; shadowed regions and whiskers show the standard deviation.


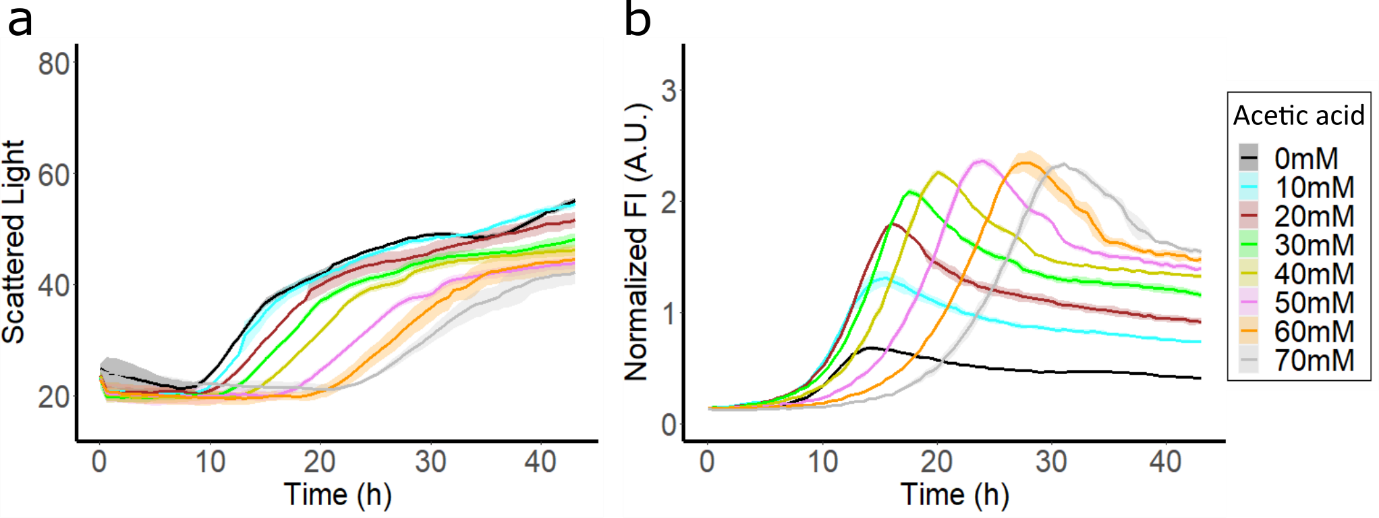


**Figure S2**. a) Growth and b) normalized fluorescence intensity of the CRISPRi library cultures expressing the biosensor (CBL) at different concentrations of acetic acid. The strains were grown in microbioreactors at pH 3.5. Data obtained from three biological replicates; shadowed regions show the standard deviation.


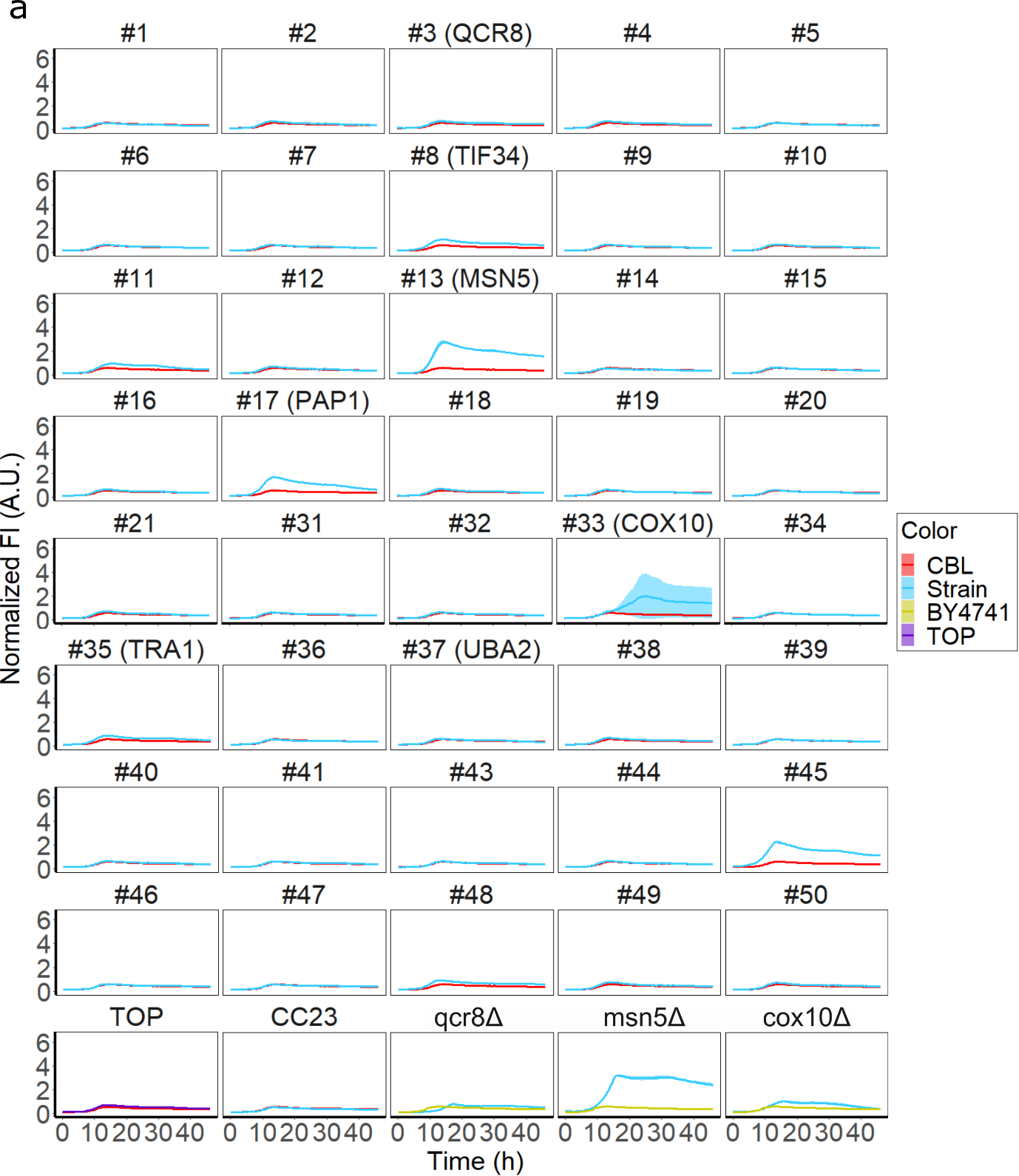

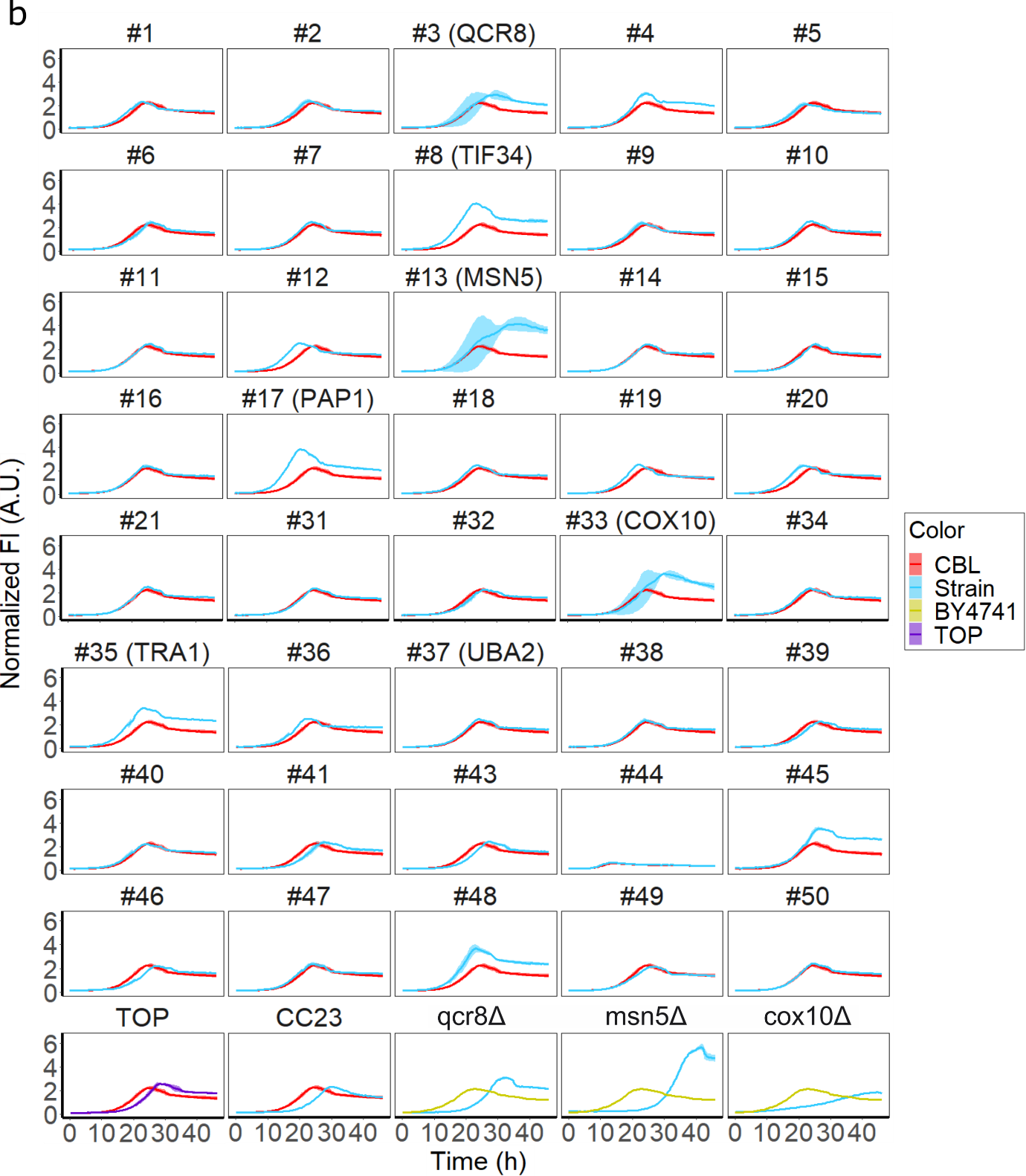

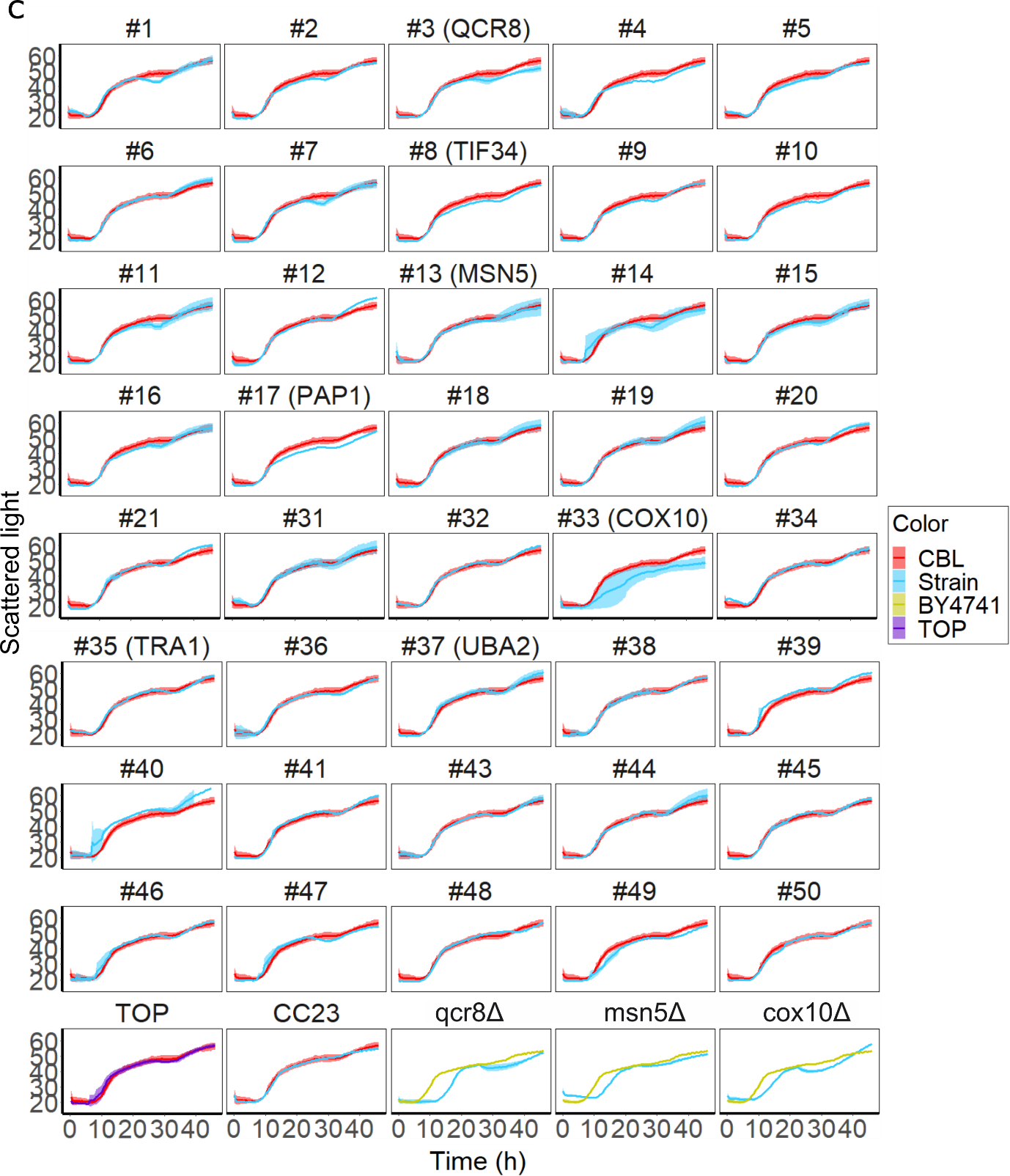

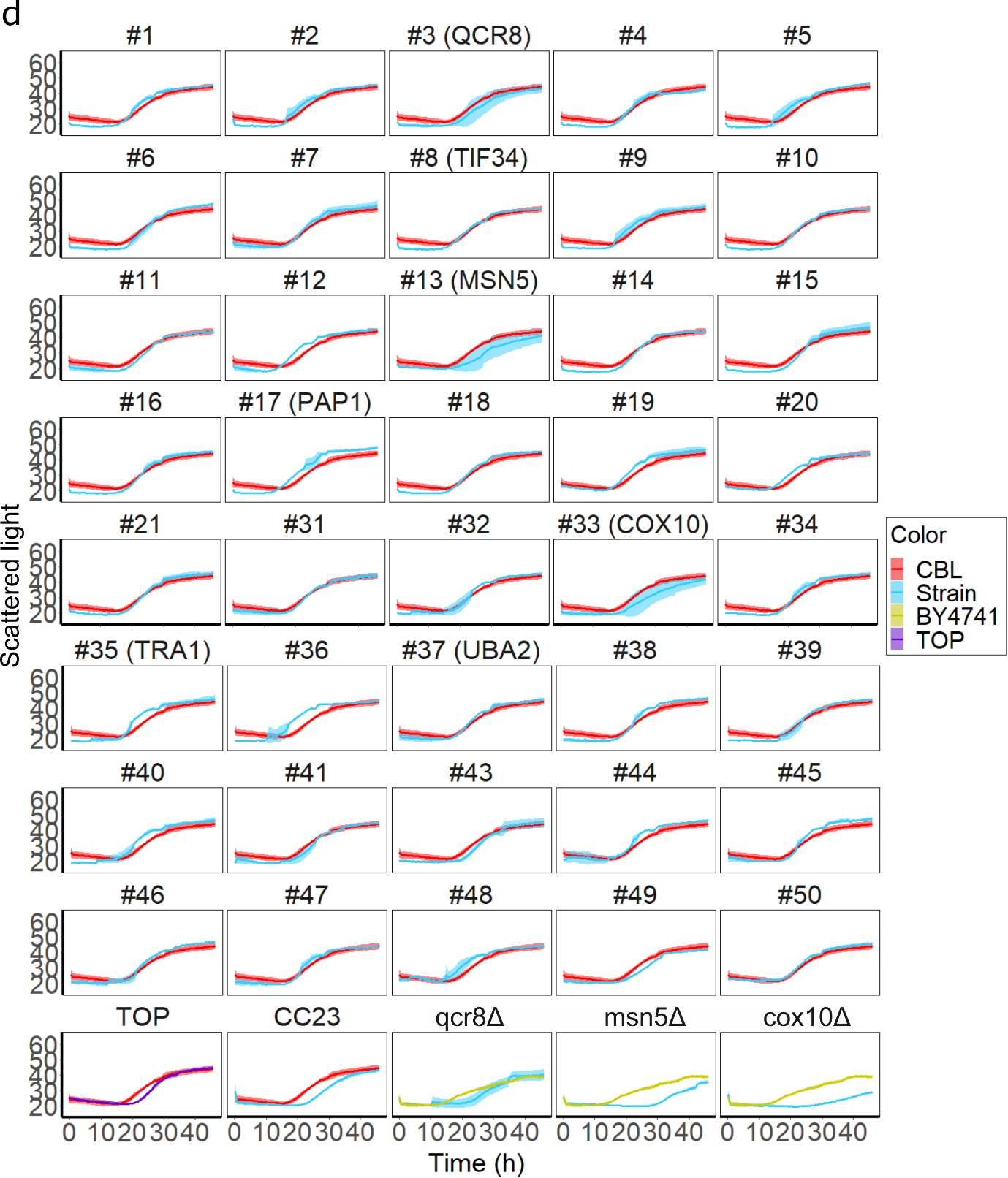


**Figure S3**. a, b) Normalized fluorescence intensity (FI) of the CBL pool (red lines), the isolated strains (green lines) and the TOP pool cultures (blue lines) at 0 (a) or 50 mM (b) acetic acid. c, d) The growth measured as scattered light of the CBL pool (red lines), the isolated strains (green lines) and the TOP pool cultures (blue lines) at 0 (a) or 50 mM (b) acetic acid. Data obtained from seven (CBL), five (strains #3, #13 and #33) four (TOP) three (deletion mutants *qcr8Δ*, *msn5Δ*, *cox10Δ* and control strains BY4741 and CC23) or two (all other samples) biological replicates; shadows show the standard deviation.


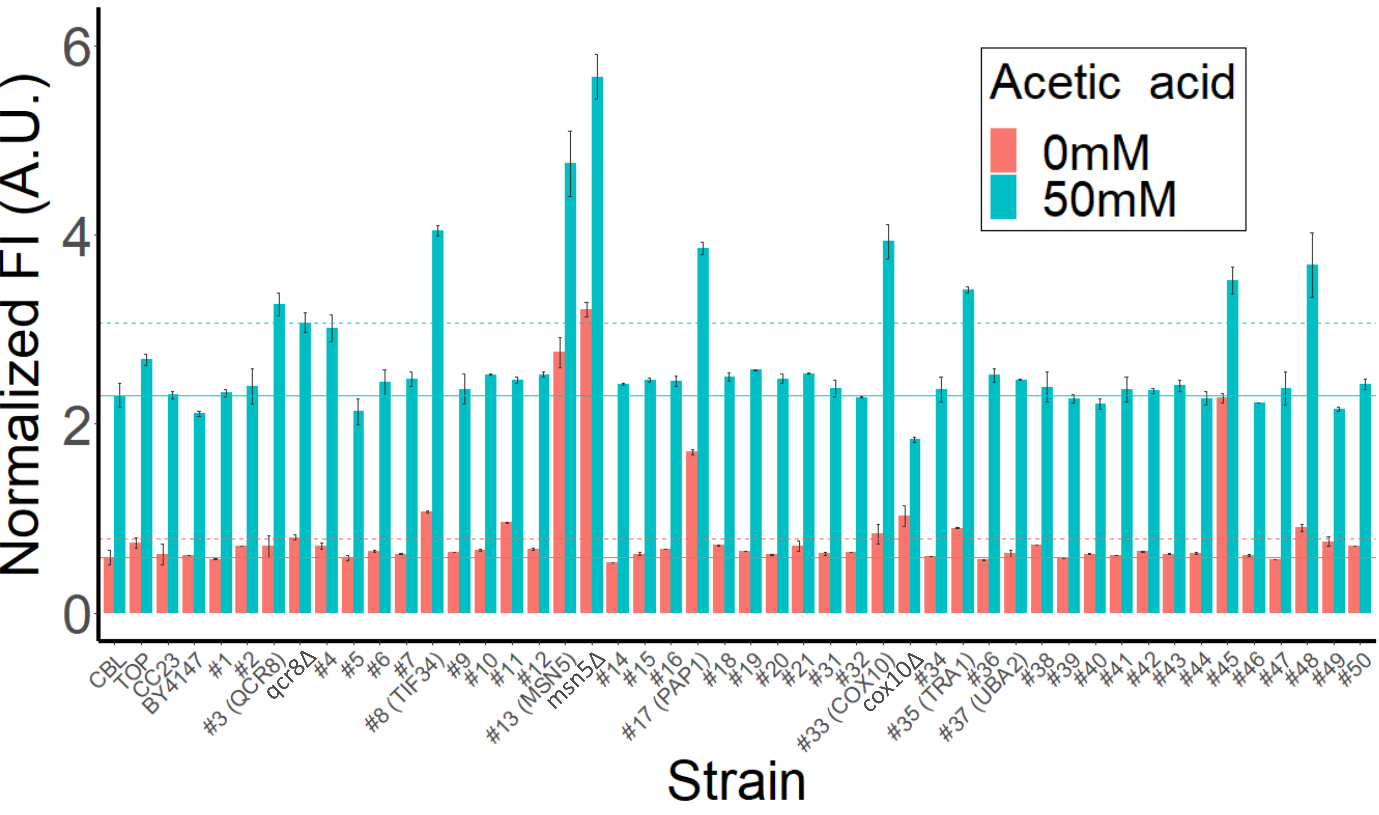
 **Figure S4**. Normalized maximal fluorescence intensity (FI) of the pooled library (CBL) and selected isolates at o and 50 mM acetic acid, at pH 3.5. Red and cyan lines show the normalized FI average value of CBL when cultivated at 0 and 50 mM acetic acid (solid lines). Dashed lines show a 33% increase in FI compared to the CBL. Data obtained from seven (CBL), five (strains #3, #13 and #33) four (TOP) three (deletion mutants *qcr8Δ*, *msn5Δ*, *cox10Δ* and control strains BY4741 and CC23) or two (all other samples) biological replicates; whiskers show the standard deviation.


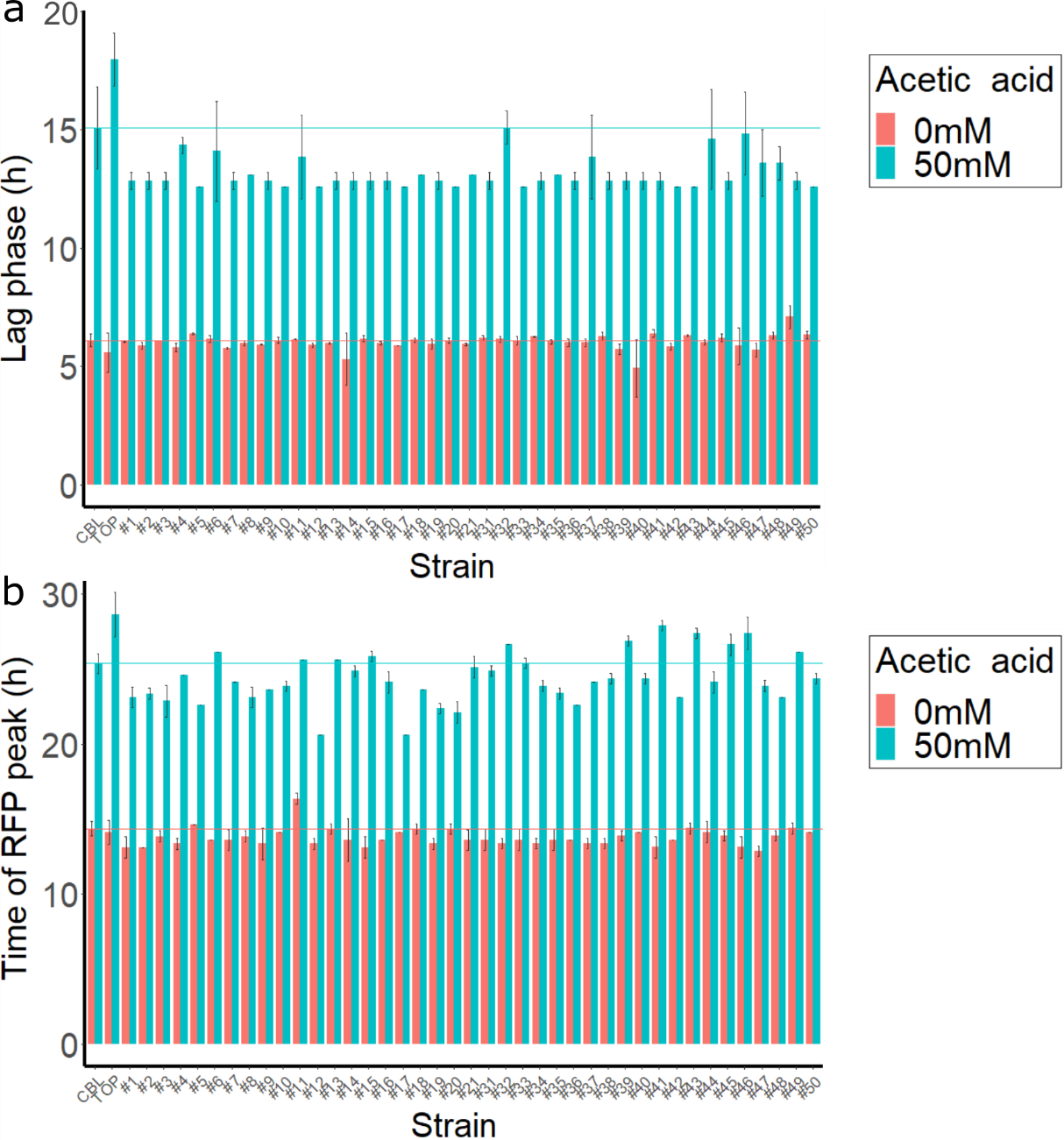


**Figure S5.** a) Length of lag phase and b) time to reach the peak in reporter expression of the CBL pool the TOP pooled and strains isolated through FACS at 0 and 50 mM acetic acid. Red and cyan lines show the value of the CBL pool. Data obtained


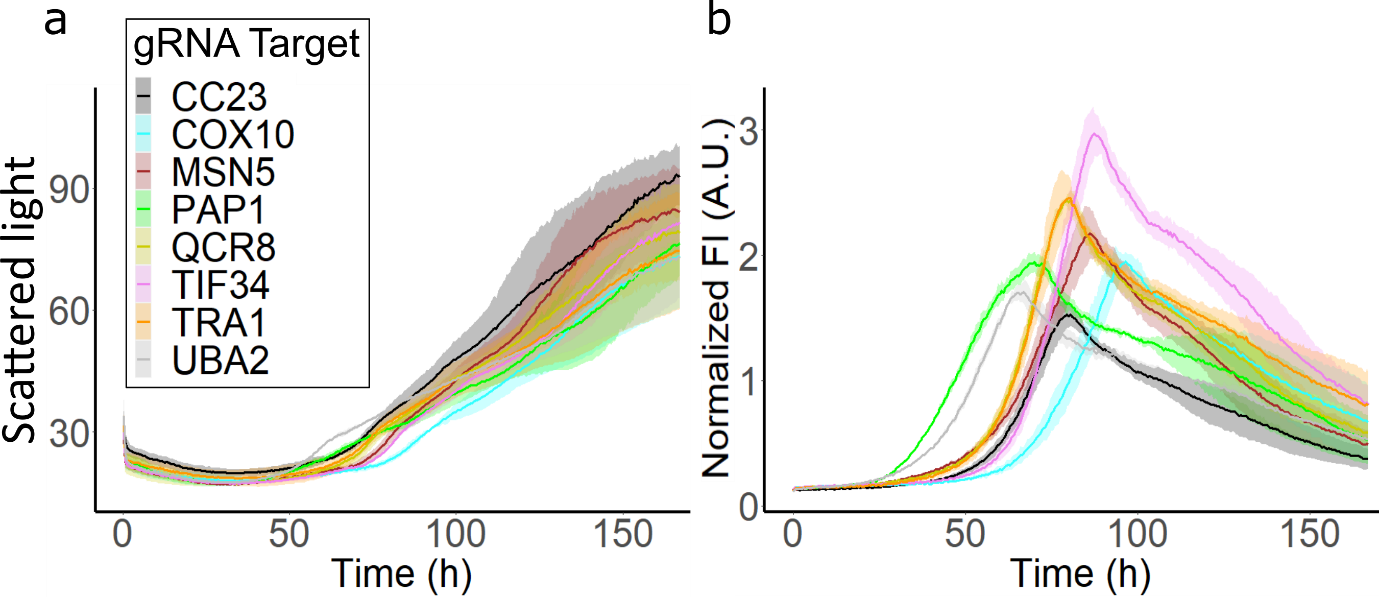


**Figure S6**. a) Growth measured as scattered light and b) normalized fluorescence intensity (FI) of selected strains at 150 mM acetic acid. The strains were grown in microbioreactors at pH 4.5. Data obtained from three biological replicates; shadowed regions show the standard deviation.
